# Interleukin-17: Potential Target for Chronic Wounds

**DOI:** 10.1155/2019/1297675

**Published:** 2019-11-18

**Authors:** Yasmin Hadian, Michelle D. Bagood, Sara E. Dahle, Apra Sood, R. Rivkah Isseroff

**Affiliations:** ^1^Department of Dermatology, School of Medicine, University of California, Davis, CA, USA; ^2^Dermatology Division, VA Northern California Health Care System, Mather, CA, USA; ^3^Podiatry Division, VA Northern California Health Care System, Mather, CA, USA

## Abstract

Chronic wounds exhibit persistent inflammation with markedly delayed healing. The significant burden of chronic wounds, which are often resistant to standard therapy, prompts further research on novel therapies. Since the interleukin-17 family has been implicated as a group of proinflammatory cytokines in immune-mediated diseases in the gut and connective tissue, as well as inflammatory skin conditions, we consider here if it may contribute to the pathogenesis of chronic wounds. In this review, we discuss the interleukin-17 family's signaling pathways and role in tissue repair. A PubMed review of the English literature on interleukin-17, wound healing, chronic wounds, and inflammatory skin conditions was conducted. Interleukin-17 family signaling is reviewed in the context of tissue repair, and preclinical and clinical studies examining its role in the skin and other organ systems are critically reviewed. The published work supports a pathologic role for interleukin-17 family members in chronic wounds, though this needs to be more conclusively proven. Clinical studies using monoclonal interleukin-17 antibodies to improve healing of chronic skin wounds have not yet been performed, and only a few studies have examined interleukin-17 family expression in chronic skin wounds. Furthermore, different interleukin-17 family members could be playing selective roles in the repair process. These studies suggest a therapeutic role for targeting interleukin-17A to promote wound healing; therefore, interleukin-17A may be a target worthy of pursuing in the near future.

## 1. Introduction

More than 9 million people in the United States are diagnosed with chronic wounds, and the incidence rate is expected to grow rapidly with the aging and increasingly diabetic and obese population [1]. Treatment for chronic wounds costs approximately $28-31 billion each year [1]. The public health concern and excessive financial burden warrant further efforts to effectively manage chronic wounds. “Chronic” wounds are defined by the US Centers for Medicare & Medicaid Services (CMS) as “wounds that do not heal completely after receiving standard medical treatment for 30 days” [2] and are characterized by delayed reepithelialization with persistent elevation of inflammatory markers [3–5]. While inflammation is a necessary component of the early wound healing process, excessive, prolonged, and dysregulated inflammation is associated with impaired wound healing [3–5]. The lack of response to standard therapies prompts ongoing studies for pathogenic biomarkers and trials for novel therapies. Therefore, in this review, we discuss the potential pathologic role of the interleukin-17 (IL-17) family in the wound repair process in order to evaluate it as a possible target for therapy.

The IL-17 family consists of a group of proinflammatory cytokines with an active role in host defense, yet implicated in the pathogenesis of a wide range of immune-mediated diseases, including psoriasis, psoriatic arthritis, rheumatoid arthritis, ankylosing spondylitis, and others such as hidradenitis suppurativa [6–9]. Members of the IL-17 family that are now recognized as critical cytokines altering skin function in psoriasis and psoriatic arthritis are IL-17A, IL-17C, and IL-17F. These cytokines act on keratinocytes to induce the expression of several chemokines leading to the recruitment and accumulation of neutrophils, T cells, and dendritic cells, causing epidermal and vascular hyperplasia as seen in psoriasis [6, 10]. Studies have revealed increased expression of IL-17A, IL-17C, and IL-17F in psoriatic skin as compared to nonlesional skin with the upregulation of IL-17A showing a positive association with disease severity [11, 12]. Monoclonal antibodies targeting IL-17A (i.e., secukinumab and ixekizumab) or the IL-17 receptor subunit IL-17RA (i.e., brodalumab) have already demonstrated dramatic therapeutic results in patients with psoriasis, psoriatic arthritis, and ankylosing spondylitis, thus confirming the pathogenic relevance of IL-17 family members in mediating inflammation in psoriasis, psoriatic arthritis, and ankylosing spondylitis [13–16].

Recent studies have also demonstrated the IL-17 family to be a possible mediator of inflammation in hidradenitis suppurativa. Hidradenitis suppurativa (HS) has shown a T-helper 17 cell- (T_H_17-) skewed cytokine profile in inflamed HS skin, based on intracellular cytokine staining, with the ratio of T_H_17 to regulatory T cells dysregulated in favor of T_H_17 cells (identified by flow cytometry using antibody to IL-17A or IL-17F). Lesional skin from antitumor necrosis factor- (TNF-) treated HS patients revealed a reduction in the frequency of T_H_17 cells and normalization of the T_H_17 to regulatory T cell ratio [17]. This clustering of T_H_1/T_H_17-associated cytokines around the lesional inflammation has also been demonstrated in other studies on HS [18].

Six members of the IL-17 family have been identified (IL-I7A, IL-17B, IL-17C, IL-17D, IL-17E/IL-25, and IL-17F) [9] by their conserved C-terminal region but differing N-terminal segments [19]. Different members of the IL-17 family have opposing signaling pathways and biological outcomes; however, not all pathways have been comprehensively elucidated. Thus, we have focused on the signaling pathways most relevant to the role of the IL-17 family members in wound pathogenesis. Furthermore, the role of the IL-17 family in chronic cutaneous wounds has not been fully determined; however, the cumulative preclinical and clinical studies in other organ systems suggest that further investigation is warranted to determine its potential as a therapeutic target for chronic wound patients.

## 2. IL-17 Pathway

**Figure 1:**
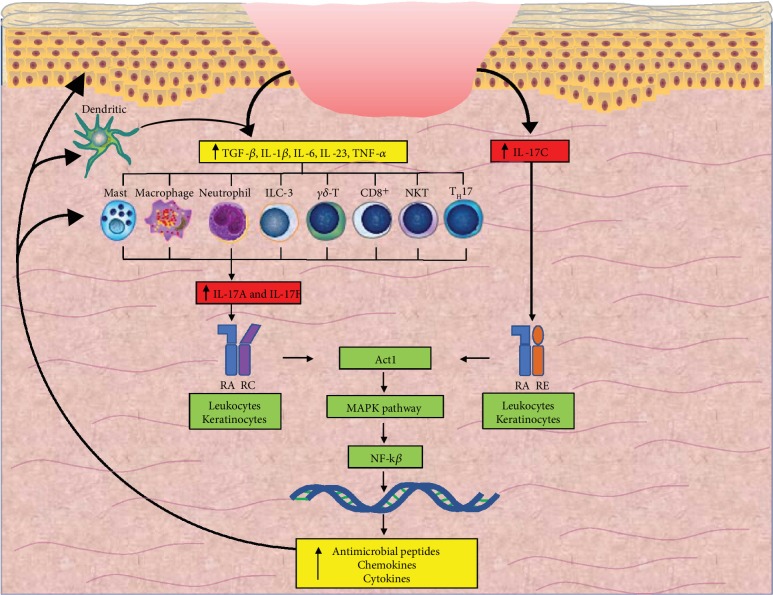
Skin wounding induces the release of IL-17A, IL-17F, and IL-17C from keratinocytes and leukocytes. These ligands bind to their respective heterodimeric receptors (IL-17A and IL-17F at IL-17RA/RC; IL-17C at IL-17RA/RE) on keratinocytes and leukocytes to release antimicrobial peptides, chemokines, and cytokines—contributing to a self-perpetuating cycle of inflammation. TGF-*β*: transforming growth factor-*β*; IL: interleukin; TNF-*α*: tumor necrosis factor-*α*; ILC3: Type 3 innate lymphoid cells; NKT: natural killer T; T_H_17: T-helper 17; RA: receptor A subunit; RC: receptor C subunit; RE: receptor E subunit; MAPK: mitogen-activated protein kinase; NF-*κ*B: nuclear factor-kB.

The members of the IL-17 family are released from cells of both the innate and adaptive arms of immunity, as well as a wide range of nonhematopoietic cells such as epithelial cells, depending on the cytokine subtype (Table 1) [9]. Transforming growth factor-*β* (TGF-*β*) and proinflammatory cytokines IL-1*β*, IL-6, and IL-23 stimulate IL-17A and IL-17F release from T-helper 17 (T_H_17) cells, CD8-positive T cells, *ɣδ*-T cells, natural killer T (NKT) cells, and Type 3 innate lymphoid cells (ILC3), while IL-1*β* and TNF-*α* activate IL-17C release from epithelial cells (Figure 1) [20–32]. Additional studies have suggested the release of IL-17A from macrophages, neutrophils, mast cells, and dendritic cells and IL-17C from mononuclear cells (Figure 1) [28, 33–38]. Interestingly, IL-17E has been shown to attenuate the T_H_17 response in a model of experimental autoimmune encephalomyelitis by stimulating the production of the T-helper 2 (T_H_2) cytokine IL-13, which inhibits the production of T_H_17-promoting cytokines IL-1*β*, IL-6, and IL-23 from dendritic cells [39]. This suggests the possibility of IL-17E as a potential downregulator of IL-17A and IL-17F.

Upon release, IL-17 family ligands bind heterodimeric transmembrane receptors consisting of a common IL-17 receptor A (IL-17RA) subunit and a unique second subunit specific for cytokine subtype: IL-17A and IL-17F bind IL-17RA/RC; IL-17C binds IL-17RA/RE; IL-17B and IL-17E bind IL-17RA/RB (Table 1) [32, 40–42]. The receptor moiety specific for IL-17D has yet to be identified. All five IL-17 receptors share a fibronectin type III (FnIII) extracellular domain and, contrary to other cytokine receptors but similar to toll-like receptors, a cytoplasmic SEF-IL-17R domain [43]. Upon binding to their receptors, IL-17A, IL-17C, and IL-17F induce a conformational change in their receptors that allows heterodimeric complex formation between the specific receptor subunits (subunits RA and RC heterodimerize upon binding ligands IL-17A or IL-17F; subunits RA and RE heterodimerize upon binding ligand IL-17C) and subsequently activates Act1, which is an adaptor protein that initiates the mitogen-activated protein kinase (MAPK) and nuclear factor-kB (NF-*κ*B) signaling cascades (Figure 1) [43–49]. These signaling pathways lead to the expression of antimicrobial peptides, chemokines, and cytokines such as TNF-*α*, IL-1*β*, IL-6, and IL-23, thereby triggering recruitment of additional proinflammatory immune cells including neutrophils and T_H_17 cells, further perpetuating the inflammation cycle (Figure 1) [50–53]. Specifically in keratinocytes, IL-17A activates a signaling cascade via the IL-17R–Act1–TRAF4–MEKK3–ERK5 pathway, which stimulates keratinocyte proliferation [54]. In addition to proliferation, primary keratinocytes stimulated with IL-17 alone transcribe several neutrophil chemokines and antimicrobial peptides, creating a proinflammatory skewed environment [55].

It is also important to note that the IL-17 family exhibits synergistic effects with other cytokines such as TNF-*α*, IL-1, and IL-6 [52, 56–59]. In contrast with other IL-17 subtypes, IL-17E has been implicated to induce the release of T_H_2-related cytokines, instigating eosinophilic recruitment in allergic and parasitic conditions [8, 9, 48]. Further studies are necessary to clarify the signaling pathway of IL-17B and IL-17D.

## 3. IL-17 Modulates Tissue Repair

### 3.1. In Skin

In homeostasis, genetic deletion of IL-17RA on nonhematopoietic cells in mice resulted in increased filaggrin monomer expression, decreased barrier integrity, increased inflammation, and skin microbiome dysbiosis [62]. This study demonstrated the regulatory role that IL-17RA signaling plays in maintaining the barrier function of uninjured skin and managing the delicate relationship between the skin microbiome and host inflammatory response. Upon tissue injury, IL-17 family members are released in the early phase of inflammation in multiple tissue types [7, 63], including the skin and joints [64, 65].

While the IL-17 family members may initially play a beneficial role in acute wound healing by promoting keratinocyte proliferation and production of antimicrobial peptides [7, 38, 54, 55], unregulated IL-17 family signaling may lead to prolonged inflammation and delayed wound healing. Tanno and colleagues found that deletion of invariant natural killer T cells (iNKTs) delayed healing via increased macrophage inflammatory protein-2 (MIP-2), keratinocyte chemoattractant, and IL-17A production, which increased neutrophil infiltration and decreased neutrophil apoptosis, suggesting that iNKTs act to limit neutrophil recruitment and dwelling to allow proper resolution of the inflammatory phase of healing [66]. In addition to proinflammatory macrophages, dermal V*γ*4 T cells produce IL-17A that acts in conjunction with IL-1*β* on dendritic epidermal T cells to inhibit IGF-1 production, resulting in delayed healing [67]. Therefore, these studies demonstrate that it is imperative to regulate the expression of IL-17A in the healing process.

Persistently elevated levels of IL-17 family members may be associated with wound chronicity. Indeed, studies have shown that levels of the IL-17 family in wound fluid from venous ulcers are elevated relative to the patients' contemporaneously sampled venous blood levels, and the IL-17 family is likewise elevated in lesional tissue samples from patients with pyoderma gangrenosum relative to normal skin [68, 69]. However, neither study examined specific subtypes of the IL-17 family. Another study demonstrated that IL-17C and its receptor are expressed by human keratinocytes from recurrent oral aphthous ulcers, whereas IL-17A and its receptor were not [70]. This finding may indicate the site-specific functional role of different IL-17 subtypes. Interestingly, a recent study found decreased IL-17A in wound fluid from chronic diabetic foot ulcers when compared to acute surgical wounds [71]. However, it is important to consider the difference not only in the timing of the wounds, since IL-17 family expression is increased in the early phase of healing as expected in an acute wound, but also the etiological differences between diabetic and surgical wounds, which may have a confounding influence when comparing these two separate cohorts. Furthermore, other IL-17 subtypes that may have exhibited different patterns were not included in the study.

Animal models have also suggested the pathologic role of the IL-17 family in wound healing (Table 2). The effects may be mediated by cellular responses, specifically that of macrophages and neutrophils. Rodero et al. found that genetic deletions in IL-17A or administration of IL-17A antibody accelerated wound healing in association with increased expression of a prohealing macrophage population [72]. Conversely, recombinant IL-17A (rIL-17A) administration to wild-type wounds produced delayed healing due to increased inflammatory cell infiltration [53]. Consistent with these findings, diabetic mice with IL-23 or IL-17 family gene deletions or blocking antibodies also demonstrated accelerated wound healing in association with alternatively activated prohealing macrophage expression [73]. Yet another study showed that injection of rIL-17, subtype not specified, into excisional wounds increased macrophage infiltration and monocyte chemotactic protein (MCP) production [74], while treatment with the IL-17 production inhibitor Y320 accelerated wound healing at earlier timepoints [74].

IL-17A may also modulate collagen formation in the wound. One study demonstrated that IL-17A knockout mice displayed enhanced wound closure and collagen deposition as well as decreased neutrophilic infiltrate when compared to wild-type mice [53]. Interestingly, adding a neutrophil elastase inhibitor to rIL-17A administration ameliorated the detrimental effects of rIL-17A, resulting in wound healing similar to wild-type mice [53]. Therefore, amplified IL-17A expression in the wound environment may promote collagen degradation by stimulating proteases secreted from neutrophils. Matrix metalloproteinase induction by the IL-17 family, alone or synergistically with IL-1*α*, IL-6, and TNF-*α*, may also contribute to extracellular matrix degradation in nonhealing wounds [44, 59, 75–78].

Furthermore, wounds in IL-23-deficient mice displayed reduced expression of the IL-17 family, which was subsequently reversed by injecting IL-23 directly into the wound bed [73]. These results highlight the relationship between the IL-17 family and IL-23, implicating the IL-17 family as a downstream molecule of IL-23.

Overall, studies investigating the expression of the IL-17 family in cutaneous wounds are scarce; however, findings of related proinflammatory cytokines that induce or synergize with the IL-17 family, such as TNF-*α*, IL-1, IL-6, and IL-23, in chronic wounds of different etiologies wounds may implicate the IL-17 family in the pathogenesis of these wounds [68, 69, 79–81].

### 3.2. In Connective Tissue

In addition to cutaneous wounds, the IL-17 family has been implicated in other types of degenerative tissue pathology. The IL-17 family was markedly expressed in synovial fluid from idiopathic juvenile arthritis patients and was positively correlated with disease severity [78]. Similarly, IL-17A and IL-17F were detected in synovial tissue from rheumatoid arthritis patients [58]. IL-17A induced a stronger response than IL-17F in downstream cytokine production in the synovium, additionally displaying synergism with TNF-*α* [58]. The stronger response from IL-17A suggests greater ligand affinity and/or efficacy than IL-17F upon binding its receptor and a potentially more effective therapeutic target than IL-17F. In a murine cortical bone defect model, blocking IL-17A improved healing even better than blocking the known bone growth regulator, the receptor activator of nuclear factor kappa-B ligand (RANKL) (Table 2) [82]. This study importantly highlights the time-dependent effects of IL-17A on tissue repair, as blocking IL-17A initially reduced bone volume three days post injury yet was associated with enhanced bone healing by days 10 and 21. Another study demonstrated increased IL-17A in the oral mucosa at tooth extraction sites of nonhealing osteonecrotic tissue and in serum of humans and mice associated with proinflammatory M1 macrophage phenotypic shift and the protective effects of blocking the IL-17 family associated with prohealing M2 macrophage phenotype in mice (Table 2) [83].

### 3.3. In Intestinal Tissue

Additionally, the IL-17 family appears to play a protective role in intestinal epithelium tissue repair [84–86]. Indeed, the increased incidence of inflammatory bowel disease (IBD) has been reported following treatment with secukinumab, ixekizumab, and brodalumab in psoriasis and ankylosing spondylitis patients, although causation has not been confirmed [87, 88]. Therefore, therapeutic agents targeting the IL-17 family in chronic wounds may not be appropriate in patients with IBD. The protective effects of the IL-17 family in the gut may parallel its protective effects in the skin by mediating the microbiome and modulating inflammation [89]. Inhibiting these similarly protective effects in the skin with anti-IL17 monoclonal antibody alone may increase the risk of delayed wound healing due to skin dysbiosis and an increased risk for wound infection. Thus, it may be prudent to use topical antimicrobial agents adjunctively with anti-IL-17 monoclonal antibody for chronic wounds that have failed to heal with standard of care alone. Clinical trials would be necessary to determine the appropriate management.

### 3.4. Proreparative Effects

Contrary to the reported antireparative effects of the IL-17 family, proreparative effects have been suggested in two studies (Table 3). Indeed, anti-IL-17 antibody administration in rats with medial collateral ligament injury did not improve healing but rather resulted in proinflammatory cytokine production, increased T lymphocytes, and decreased prohealing macrophage phenotype expression [90]. The experimental model consisted of male rats at an unspecified age range, not accounting for the effects of aging and sexual dimorphism on wound healing and T_H_17/IL-17 expression or function [91–99]. Furthermore, the data was collected at only one timepoint, seven days post injury, limiting examination of the kinetics of specific IL-17 subtypes. Additionally, the effects of the IL-17 family in ligamentous injury may differ from cutaneous wounds due to histological differences. Proreparative effects of IL-17A in cutaneous wounds were suggested when Macleod et al. demonstrated that pharmacological blocking or genetic deletions of IL-17A resulted in delayed wound closure in mice, in contrast with findings reported by other aforementioned animal studies on cutaneous wounds [38]. Administering rIL-17A improved wound healing in IL-17A^−/−^ mice, highlighting the beneficial role of IL-17A in the early stages of healing [38]. The inconsistent findings may indicate different roles of IL-17A in different age groups or genders, as Macleod et al. did not account for either. Time of administration relative to the stage of healing may also skew results. As age and gender may influence wound healing and T_H_17/IL-17 expression or function, it is imperative for studies to match subjects by these confounding variables and assess differences among groups.

## 4. Adverse Events of Targeting the IL-17 Family

If clinical trials targeting the IL-17 family were to be performed for chronic wound management, then it would be prudent to consider previously reported adverse events. In addition to the increased incidence of IBD that we have discussed, other adverse events that have been reported at relatively low incidence with targeting the IL-17 family include neutropenia, noncutaneous infection, and depression with or without suicidality, although causality of the latter has been recently disproven [100–102]. Since chronic skin wounds often display increased relative abundance of pathogenic bacteria *Staphylococcus aureus* and *Pseudomonas aeruginosa* in wound tissue, neutropenia and potential dissemination of infection are of particular concern [103–105]. Thus, monitoring for neutropenia in wound patients receiving monoclonal IL-17 antibody therapy may be warranted.

## 5. Conclusion

The search for chronic wound biomarkers and therapeutic targets is critical due to the growing numbers of patients suffering from this ailment. The role of the IL-17 family members in cutaneous disease indicated its potential involvement in the pathogenesis of chronic wounds. In our review of the literature, we found animal studies demonstrating a proreparative role for IL-17A in cutaneous wound healing in tissue homeostasis and in response to acute wound healing. However, the IL-17 family appears to play a role in the pathogenesis of chronic wounds (summarized in Figure 1). Studies in animal wound models with age-matched and gender-matched groups suggest a therapeutic role for targeting IL-17A to promote wound healing. The limited number of human studies prompts the need for assessing IL-17 family expression in a greater number of subjects with wounds of various etiological types (i.e., chronic venous ulcer, chronic diabetic ulcer, nonhealing surgical wound, and chronic burn injury). Clinical trials of monoclonal IL-17 antibody have yet to be initiated and the possibility of disseminated infection in the context of an increased microbial load from chronic wounds considered. Furthermore, expanded information on the signaling activities and biological function of each IL-17 subtype could pave the road to therapeutic interventions selectively targeting specific subtypes in order to optimize wound healing.

## Figures and Tables

**Table 1:** IL-17 family.

Subtype	Reported biological sources	Heterodimer receptor subunits
A	Mast cells, macrophages, neutrophils, ILC3, *ɣδ*-T, NKT, CD8^+^, T_H_17 [9]	RA/RC [9]
B	Gastrointestinal tract, pancreas, gonadal tissue, chondrocytes, synovial membrane, neurons, lymphocytes [40, 60]	RA/RB [40]
C	Epithelial cells in the colon, trachea, and skin [32]	RA/RE [32]
D	Pancreas, adipose, skeletal muscle, brain, heart, lung [61]	Unidentified
E	Mast cells, macrophages, eosinophils, basophils, respiratory epithelium, and mucosa [9]	RA/RB [9]
F	Mast cells, macrophages, neutrophils, ILC3, *ɣδ*-T, NKT, CD8^+^, T_H_17 [9]	RA/RC [9]

**Table 2:** Studies suggesting antireparative effects of the IL-17 family.

Disease model	IL-17 related finding	Ref.
IL-17A KO mice on C57BL/6 background full-thickness excisional wound healing, males and females	KO mice showed increased myofibroblast numbers and mature collagen with decreased neutrophil infiltration. Delayed healing generated by rIL-17A administration was improved with the addition of neutrophil elastase inhibitor.	[53]
Ob/Ob wound healing, males and females	Higher proportion of proinflammatory macrophages to proreparative macrophages in Ob/Ob wounds compared to WT results in delayed wound healing. IL-17A production by proinflammatory macrophages contributed to the delay and was ameliorated with anti-IL-17 antibody.	[72]
Ob/Ob wound healing, males and females	Blocking the IL-17A pathway improved wound reepithelialization in Ob/Ob impaired healing model. Further, obese mice with genetic IL-17 family knockout showed reduced proinflammatory macrophages and iNOS but kept proreparative macrophages that express CD206 and LYVE1.	[73]
BALB/c full-thickness excisional wound healing, males	Subcutaneous injection of recombinant mouse IL-17 family resulted in increased low-Ly6C macrophage infiltration via proinflammatory mediator levels including MCPs, which produced more type I collagen and delayed wound closure.	[74]
BALB/c bone healing, females	Drill injury to the femur in normal and OVX mice showed inhibited healing with anti-IL-17 treatment on day 3 post injury. However, this effect was reversed on days 10 and 21, when groups treated with anti-IL-17 had better healing, especially OVX mice on day 21 post injury. The impact of the IL-17 family on healing was mediated by decreased osteogenic protein expression and increased oxidative stress at the injury site.	[82]
BRONJ	BRONJ lesions showed increased IL-17^+^ cells and IL-17 family in C57BL/6 mice and humans. Lesions also showed increased M1 macrophages, which was attenuated with anti-IL-17A treatment. Exogenous IL-17, subtype not specified, enhanced M1 phenotype and suppressed M2 signaling in murine and human monocytes cultured under polarizing conditions.	[83]

KO: knockout; rIL-17: recombinant interleukin-17; Ob/Ob: obese diabetic; WT: wild type; iNOS: inducible nitric oxide synthase; MCP: monocytic chemotactic protein; OVX: osteopenic; BRONJ: bisphosphonate-related osteonecrosis of the jaw.

**Table 3:** Studies suggesting proreparative effects of the IL-17 family.

Disease model	IL-17 related finding	Ref.
C57BL/6, Tcrd^−/−^, IL-17A^−/−^ full-thickness excisional wound healing	Found an IL-17A producing subset of dendritic epidermal T cells that activate after skin injury. Further showed in vitro that exogenous IL-17A induced keratinocytes to produce antimicrobial peptides important for wound healing.	[38]
Rat ligament healing, males	IL-17, subtype not specified, neutralizing antibody treatment decreased M2 macrophage numbers and increased T cell-activating cytokines (IL-2, IL-6, and IL-12) as well as T cell numbers. Type I collagen levels were decreased but no changes were found in the wound area or length.	[90]

IL: interleukin.
